# Supplementary material for: A nanoscale photonic thermal transistor for sub-second heat flow switching
Source: Nat Commun. 2024 Jul 3;15:5584. doi: 10.1038/s41467-024-49936-0 (PMC11222488; doi:10.1038/s41467-024-49936-0)
Supplement: Supplementary file 1 — Supplementary Information [file 41467_2024_49936_MOESM1_ESM.pdf]

Supplementary Information for

A Nanoscale Photonic Thermal Transistor for Sub-Second Heat Flow Switching

Ju Won Lim[1†], Ayan Majumder[2†], Rohith Mittapally[2], Audrey-Rose Gutierrez[3], Yuxuan Luan[2], Edgar Meyhofer[,2,4] and Pramod Reddy[*,1,2,3]*

[1]Department of Materials Science and Engineering, University of Michigan, Ann Arbor, MI 48109, USA

[2]Department of Mechanical Engineering, University of Michigan, Ann Arbor, MI 48109, USA

[3]Department of Electrical Engineering and Computer Science, University of Michigan, Ann Arbor, MI 48109, USA

[4]Department of Biomedical Engineering, University of Michigan, Ann Arbor, MI 48109, USA

[†]These authors contributed equally to this paper.

[*]Email: meyhofer@umich.edu, pramodr@umich.edu

Supplementary Note 1. Fabrication of the suspended source and drain devices

The micro-fabrication processes for the source and drain devices are reported in detail elsewhere[1]. Briefly, the fabrication process, in the cross-sectional view, is illustrated in Supplementary Figure 1. Starting with a 500 µm-thick, double-side polished silicon (Si) wafer (Step 1), LPCVD low-stress silicon nitride (SiN) films with a thickness of 250 nm were deposited on both sides of the wafer (Step 2). Then, using liftoff processes (Step 3), 30 nm-thick platinum (Pt) heater-thermometer lines, followed by 100 nm-thick gold (Au) contact pads, were patterned on the top side of the wafer. Next, the SiN layers on the frontside and backside of the wafer were lithographically etched using reactive ion etching (RIE) to form a suspended structure (Step 4). The source and drain membranes were released through a final etching process using KOH (Step 5). The shield used to prevent any heat exchange between beams is not included in this schematic.

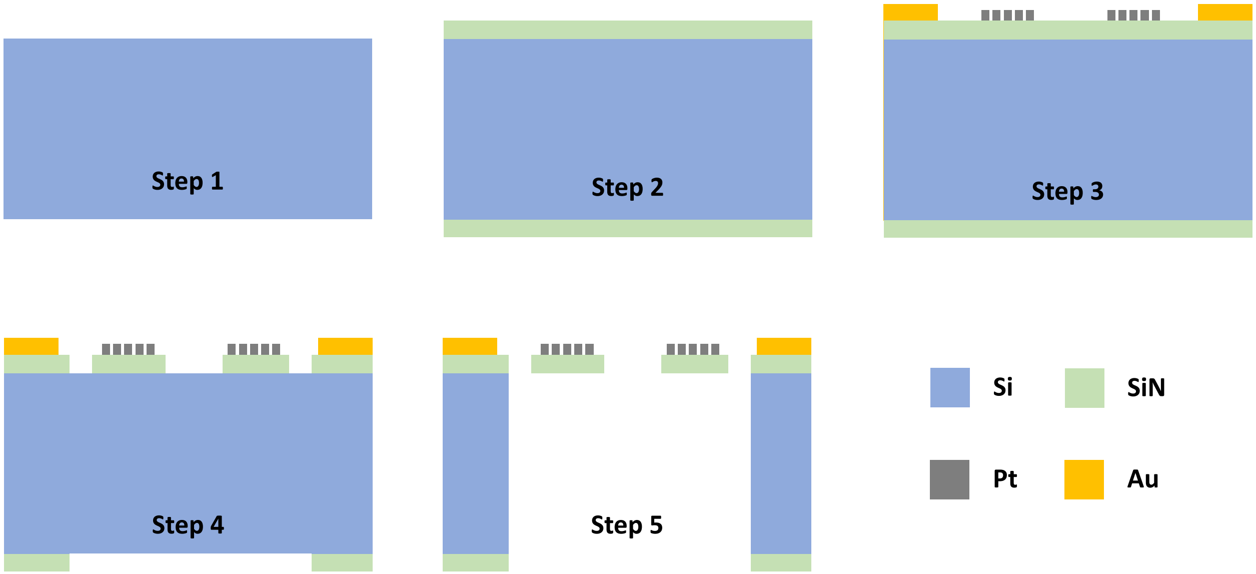

Supplementary Figure 1. Fabrication process for the suspended source and drain devices.

Supplementary Note 2. Fabrication of gate device

The fabrication process for the gate device is illustrated in Supplementary Figure 2. A *p*-doped double-bonded silicon-on-insulator (SOI) wafer (Ultrasil LLC) with a 20 ± 1 µm-thick top layer, 40 ± 1 µm-thick middle layer, 400 ± 5 µm-thick substrate, and two 1 µm-thick buried oxide layers (BOX) is chosen (Step 1). The specified resistivity of both the top and middle layers is < 0.02 Ω cm. BOX layers are used as etch stops to fabricate the suspended gate device. The top device layer is patterned and etched to form a 16 µm-tall mesa using a deep reactive ion etching (DRIE) process (Step 2). The ~4 µm-tall silicon serpentine structure is then patterned using standard lithographic techniques and etched using DRIE (Step 3). Next, a 200 nm-thick gold layer is deposited to form electrical contacts and patterns formed using a lift-off process (step 4). Note that Ti/Pt (10/30 nm) are deposited (not illustrated in step 4) underneath the Au for better contact. The device beams and the structure are patterned and etched from the top using the DRIE process (Step 5). The Si handle layer, two BOX layers, and Si middle layer are etched away from the back side of the device to release the suspended beam structure using the DRIE processes (Step 6). Subsequently, a layer of Al_2O_3 with a thickness of 10 nm was deposited onto the gate device using atomic layer deposition, serving the purpose of creating an electrically insulating layer (Step 7). Finally, a 150 nm-thick VO_x is deposited on the mesa as a phase transition material, followed by annealing at 350 °C for 5 min under an N_2 atmosphere (Step 8).

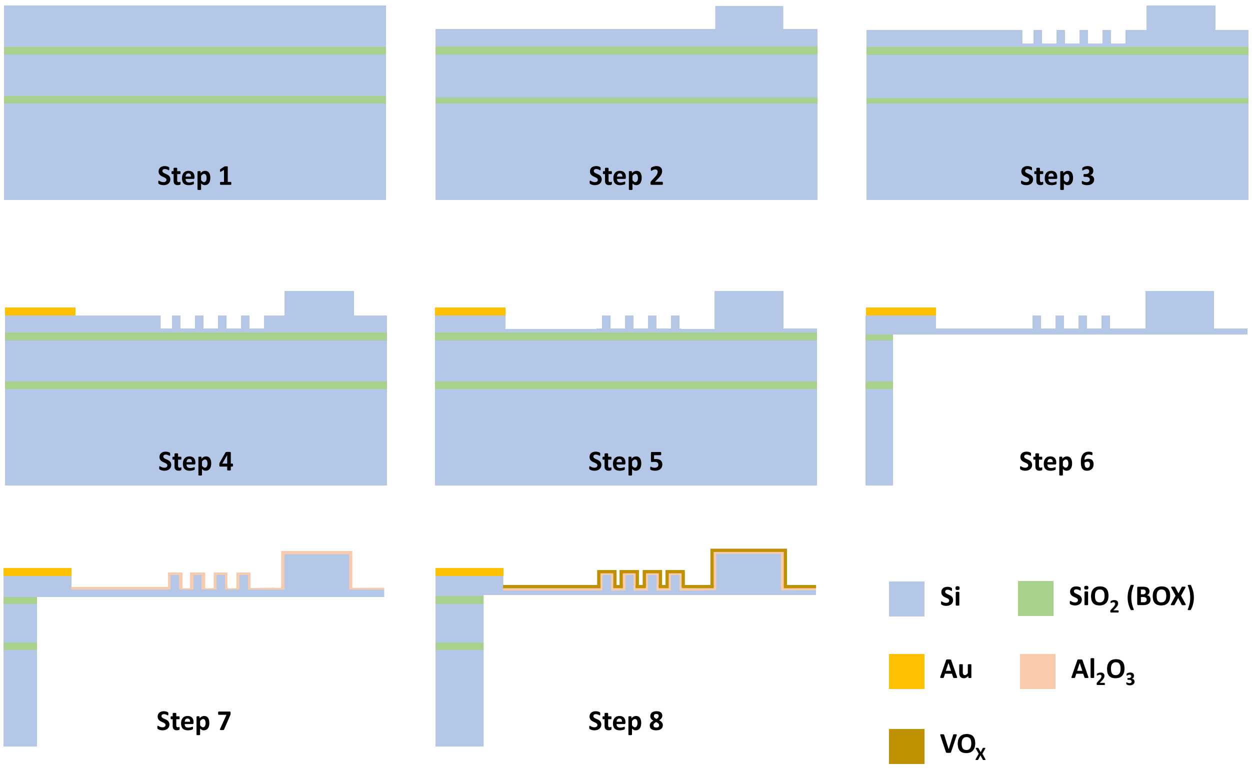

Supplementary Figure 2. Fabrication process for the gate device.

Supplementary Note 3. Deposition of vanadium oxide (VO_x) thin film

In this study, we grew a VO_x thin film on a gate device by physical vapor deposition (PVD) using a DC-pulsed magnetron sputtering process in a LAB18 sputtering system (Kurt J. Lesker Company) using a 99.9% pure vanadium target with a diameter of 3 inches and a thickness of 0.125 inches. The substrate temperature was maintained at room temperature. In the process, the DC power was set to 180 W, and both argon and oxygen were introduced into the chamber at flow rates of 48.5 sccm and 1.5 sccm, respectively. The sputtering rate of the VO_x thin film was determined to be approximately 5 nm/min by measuring the thickness of the as-deposited film. Subsequently, the sputtered VO_x thin film underwent annealing at 350 °C for 5 minutes under an N_2 ambient environment using the Jetfirst 150 RTP tool.

To evaluate the resistance of the VO_x thin film, a 150 nm-thick film was deposited on a silicon substrate. The resistance of the VO_x film was measured during the heating and cooling process, and the results are presented in Supplementary Figure 3. The observed hysteresis during the phase transition is attributed to the strain, doping, and lattice defects inside the deposited VO_x thin film[2].

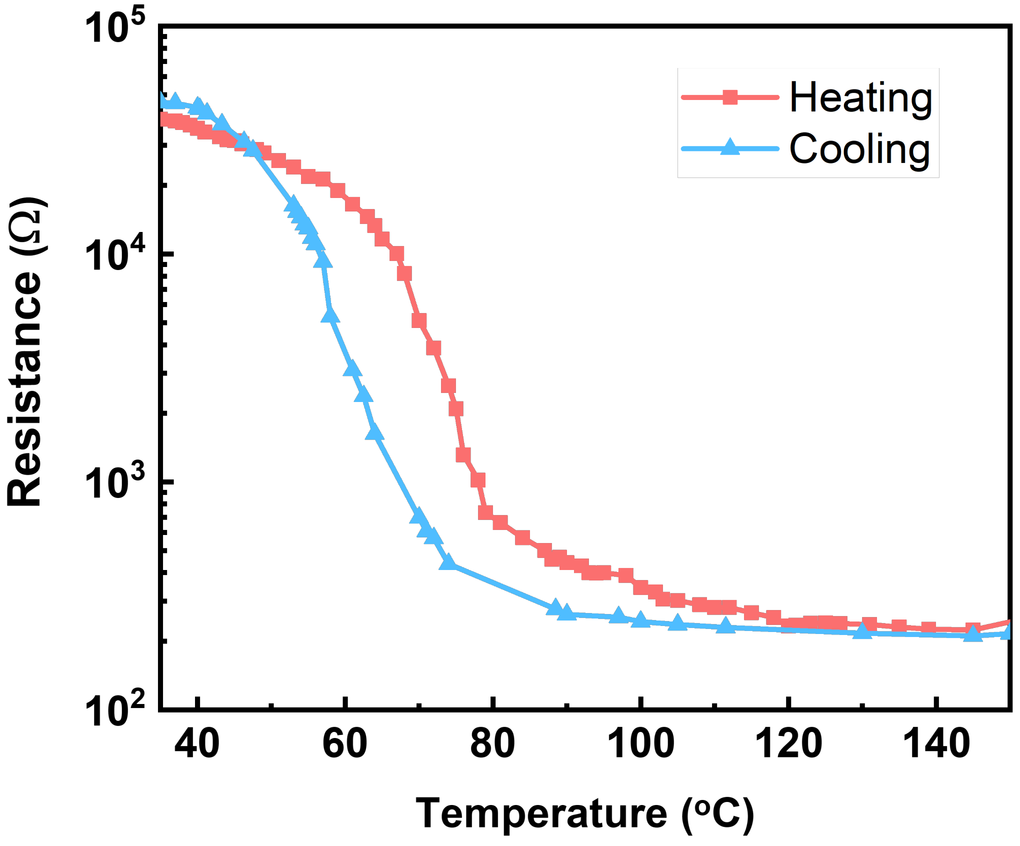

Supplementary Figure 3. Resistance characteristics of a VO_x film deposited on a bare Si as a function of the substrate temperature.

Supplementary Note 4. Effect of Si thickness on gate device in the SCUFF-EM model.

To support the choice of a 10 µm-thick Si gate for the radiative conductance and Poynting flux calculations presented in Figures 2d and 4 of the main manuscript, we examined the effect of the gate thickness on our SCUFF-EM calculations. We began by creating models with three different gate thicknesses: 5 µm, 10 µm, and 15 µm, all made from doped Si, while maintaining the source and drain SiN membranes at 80 µm long, 60 µm wide, 250 nm thick, and separated by 20 µm. All three gates were coated with 150 nm of VO_x on all sides. The three cases are schematically shown in Supplementary Figures 4a−c below.

We then computed the radiative conductance between the source and drain membranes. The gap of the gate to the source and drain SiN membranes was calculated for gap sizes (d) ranging from $d = 1$ µm to $d = 25$ µm, with the VO_x layer covering the whole surface of Si gate. Supplementary Figure 4d and Supplementary Figure 4e display the results of the gap-dependent radiative conductance at three different gate thicknesses when VO_x layer is in the insulating and metallic phases, respectively. Results indicate that the radiative conductance is independent of the Si gate thickness, specifically within the range of 5 µm to 15 µm. This implies that the VO_x layer at the bottom of the gate plays little to no role in the heat flow between source and drain when the Si thickness of the gate is 5-15 µm. Consequently, choosing a 10 µm thick Si gate with VO_x covered on all sides serves as a representative model for our experiments. Note that, in our experiments, only the top surface of the Si gate is covered by the VO_x thin film. We have excluded the consideration of the VO_x film on the sides in this argument, as we believe it plays a negligible role in the heat flow.

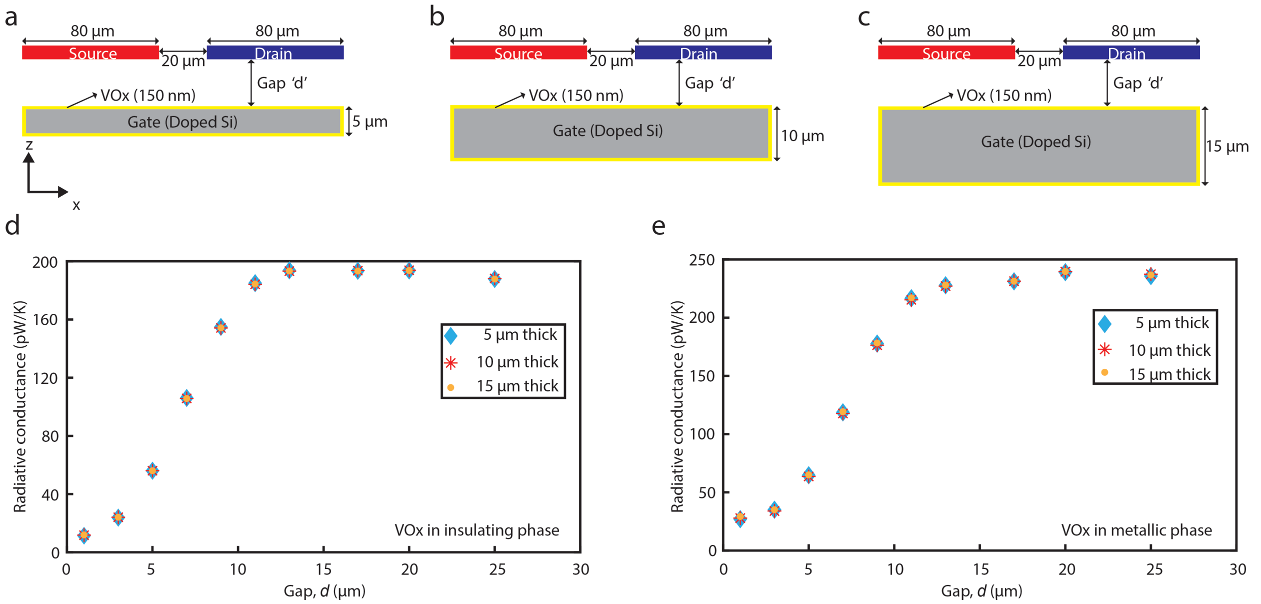

Supplementary Figure 4. Demonstration of gate thickness independence for SCUFF-EM calculations. **a,** Schematic of the model used in the calculations. **b,** Same as case a, but with the doped Si gate thickness changed to 10 µm. **c,** Same as case a but with the doped Si gate thickness changed to 15 µm. **d,** Radiative conductance calculation for cases a, b, and c with VO_x in the insulating phase. **e,** Same as d, but with VO_x in metallic phase.

Supplementary Note 5. Electrical characteristics of the source and drain devices

To characterize the thermal properties of the source and drain devices, we employed two half-Wheatstone bridges, as shown in Supplementary Figure 5a. An alternating current (I_{AC}) was applied to the half-Wheatstone bridge. A potentiometer, R_{mat}, was connected in series with the Pt serpentine line integrated into the device (R_S for source and R_D for drain) to balance the circuit. To characterize the thermal conductance of the source and drain devices, we used two-stage amplification with a gain of 1 for all three instrumentation amplifiers (AD524) to achieve a good common mode rejection. The output of the circuit was continuously monitored during the experiment using a lock-in amplifier (SR 830, Stanford Research System).

We first characterized the electrical properties of the Pt resistance thermometer integrated into the source device. The source was placed on the cold finger of the cryostat (Janis ST-100), whose temperature was controlled by the Lakeshore 335 temperature controller under a vacuum level of less than 10^{-3} Torr. At each temperature, the resistance of the Pt serpentine was measured by applying an alternating current (I_{AC} = 1 μA) through the serpentine at a frequency of 101 Hz using a Keithley 6221 current source, ensuring negligible self-heating. At the same frequency (101 Hz), the voltage (V_f) across the Pt resistance thermometer (PRT) was measured using a four-probe scheme with an SR830 lock-in amplifier to determine the resistance. The measured resistance within the temperature range of 299 K to 310 K is shown in Supplementary Figure 5b, and the measured slope (dR/dT) was found to be 29.50 ± 0.138 Ω/K with good linearity for small temperature differences. The corresponding TCR was calculated as 1.75×10^{-3} K^{-1} using the expression: $\alpha = (1/R)\times dR/dT$ (measured resistance of the source device is ~16.857 kΩ at 25 °C).

Next, we measured (G_{beams}) the thermal conductance of the source device using a 3ω-measurement method by applying a sinusoidal current with a fixed frequency of 1 Hz to the platinum resistor of the source device. The current amplitude (I_{AC}) is adjusted in steps (using Keithley 6221), ranging from 6 μA to 12 μA, to generate a temperature modulation at 2 Hz. To measure the temperature change, the corresponding V_{3f} was measured using an SR830 lock-in amplifier while various I_{AC} were applied. The resulting temperature change (ΔT_{2f}) of the source device is calculated using the following equation:

$$\Delta T_{2f} = \frac{2\Delta V_{3f}}{I_{AC} R \alpha} \quad (1)$$

Supplementary Figure 5c illustrates the power dissipation at the PRT ($Q_{2f}=I_{AC}^2R/2$) as a function of temperature rise on the source. A clear linear correlation is observed in the examined temperature range. The slope of this line corresponds to the beam thermal conductance of the source device, which is measured to be 250 ± 3 nW/K. For the drain device, electrical characteristics are almost identical to those of the source device since they were fabricated under the exact same procedure. Using the same method employed for measuring the beam thermal conductance of the source device, the measured beam conductance of the drain is determined to be 249 ± 2 nW/K. We note that G_{beams} is almost identical to G_{Th} when the gap size between the gate device and the top device is large but these two quantities differ from each other for smaller gaps.

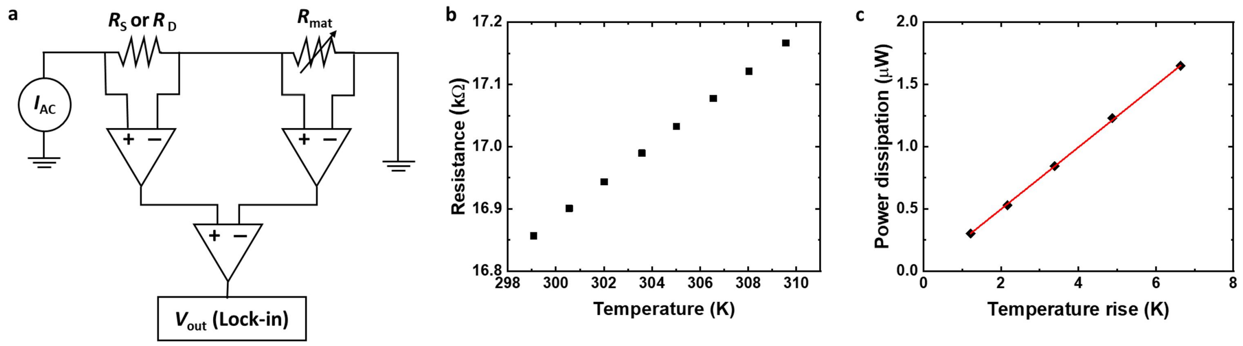

Supplementary Figure 5. Characterization of the electrical and thermal properties of the source device. **a,** Schematic of the electronic circuit used to measure temperature changes by monitoring resistance change. **b,** The measured temperature dependence of the integrated Pt resistor near room temperature. **c,** The relationship between temperature rise on the source device and the power dissipation, produced by the Pt heater, is illustrated. The measured beam conductance is represented by the slope of the red solid line.

Supplementary Note 6. Characterization of a gate device coated with a 150 nm-thick VO_x layer: Estimate of the gate device temperature and thermal conductance (G_{Gate})

Due to the significant temperature change of the gate (ΔT_g ~100 K) in comparison to the source (ΔT_s ~5 K) during experiments, the linear approximation (where $\alpha\Delta T$<<1) is not applicable, since the resistance does not vary linearly with respect to temperature. To determine the temperature of the gate, thermal conductance of the gate (G_{Gate}) needs to be characterized.

To characterize the thermal conductance of the gate, we estimated the temperature change based on the applied power by considering the resistance change. First, we measured the resistance while varying the temperature, as shown in Supplementary Figure 6a. This was done by applying a low amplitude current (fixed I_{AC} = 10 μA) at high frequency (f = 101 Hz) to eliminate self-heating while the temperature was adjusted inside a cryostat (Janis ST-100) under vacuum conditions (less than 10^{-3} Torr). Next, we obtained the resistance change as a function of dissipated power by applying a bipolar voltage (ranging from 0 V to ± 4.0 V with an E3631A power supply) to the gate under vacuum conditions (Supplementary Figure 6b). The resistance was determined from the measured current (using a 34401A digital multimeter) through the application of Ohm's law, as depicted in the inset of Supplementary Figure 6b. From these measurements, by comparing the resistance of the gate shown in Supplementary Figure 6a and Supplementary Figure 6b, we obtained the temperature as a function of the given power (Supplementary Figure 6c). The measured temperature of the gate at various bipolar voltage is as follows: 25 °C at 0 V, 33 °C at ±1.0 V, 54 °C at ±2.0 V, 68 °C at ±2.5 V, 83 °C at ±3.0 V, 100 °C at ±3.5 V, and 117 °C at ±4.0 V, respectively, with a temperature uncertainty of <±1 K for each measurement. The derivative of power (in mW) with respect to temperature (in K), indicates the total thermal conductance of the gate.

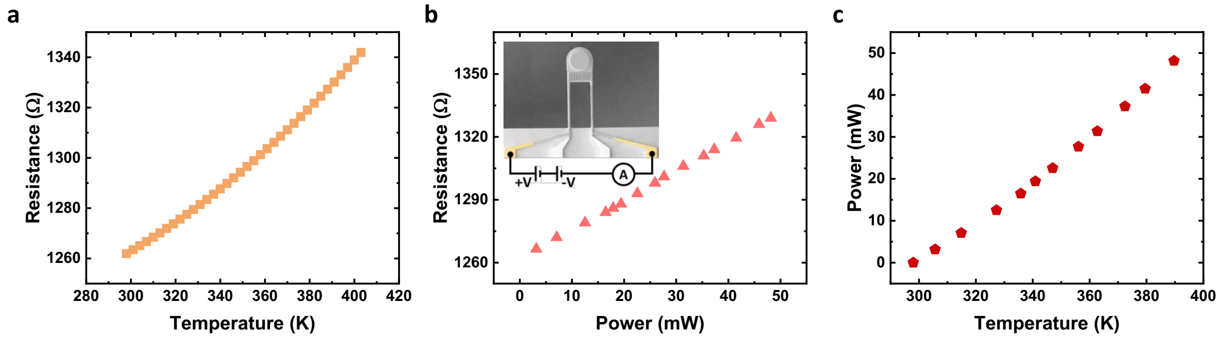

Supplementary Figure 6. Relationship between resistance, power, and temperature of a gate device. **a,** Measured resistance of the gate as a function of temperature. **b,** Measured resistance of the gate as a function of the applied power. Inset displays a false-colored SEM image demonstrating electrical connections during the measurement. **c,** A relation between the consumed power and temperature change.

Supplementary Note 7. Finite Element Method (FEM) simulations used to compute radiative conductance in the blackbody limit.

We compare our experimental results, which benefit from the presence of confinement of modes, to the blackbody limit by simulating the radiative conductance ($G_{\mathrm{blackbody}}$) between adjacent membranes using the COMSOL Multiphysics platform (AltaSim Technologies). COMSOL is able to model the electrical heating and heat transfer using the geometry and material parameters of the devices, including the platinum resistive thermometer, the beams, and the VO_x gate (see Supplementary Figures 7a−d for representative images of the COMSOL model). Unless otherwise specified, the default material parameters available in COMSOL were used. In our simulations, the ends of the beams, which are affixed to a silicon substrate in the actual devices, were given a 300 K fixed-temperature boundary condition, as was the underside of the VO_x substrate, which was resting on a heat sink in the experiment. A fixed small voltage was applied to the serpentine heater on the source membrane and two of the support beams, which induced Joule heating. The surfaces of all bodies were assigned an emissivity of 1, since they are assumed to be all blackbodies. Note the thermal conductivity used for SiN in our simulation was 3 W/(m·K) to be consistent with our measured beam conductance.

To determine the radiative conductance $G_{\mathrm{blackbody}}$ at different gap sizes, we first used the COMSOL model (shown in Supplementary Figure 7b, the model is discretized into a trapezoidal mesh of >60000 domain elements) to obtain the temperature rise in the source (ΔT_S) and the drain (ΔT_D) at different gap sizes. Next, we calculated G_{Th} (G_{Th}, total thermal conductance of the membrane device) using $G_{\mathrm{Th}} = P_{\mathrm{Joule}}/\Delta T_S$ at each gap size (see method section in the manuscript for details). With these known values, the radiative conductance ($G_{\mathrm{blackbody}}$) was computed as $G_{\mathrm{blackbody}} = G_{\mathrm{Th}}\Delta T_D/(\Delta T_S - \Delta T_D)$. For gap sizes between the source-drain device and the gate device of 0.5, 10, and 24 µm, the radiative conductance ($G_{\mathrm{blackbody}}$) is nearly identical (~4 pW/K), regardless of the phase of VO_x. Our experimental result and this simulation result are in good agreement with recent experimental findings that a hundred-fold enhancement in far-field radiative heat transfer over the blackbody limit can be achieved when the dimensions of objects are smaller than subwavelength dimensions[3]. Note that the FEM simulation results (see Supplementary Figure 7d) also exhibit good temperature uniformity (<0.1% temperature difference) on the area of the suspended membranes as well as a linear change of the temperature along the support beams.

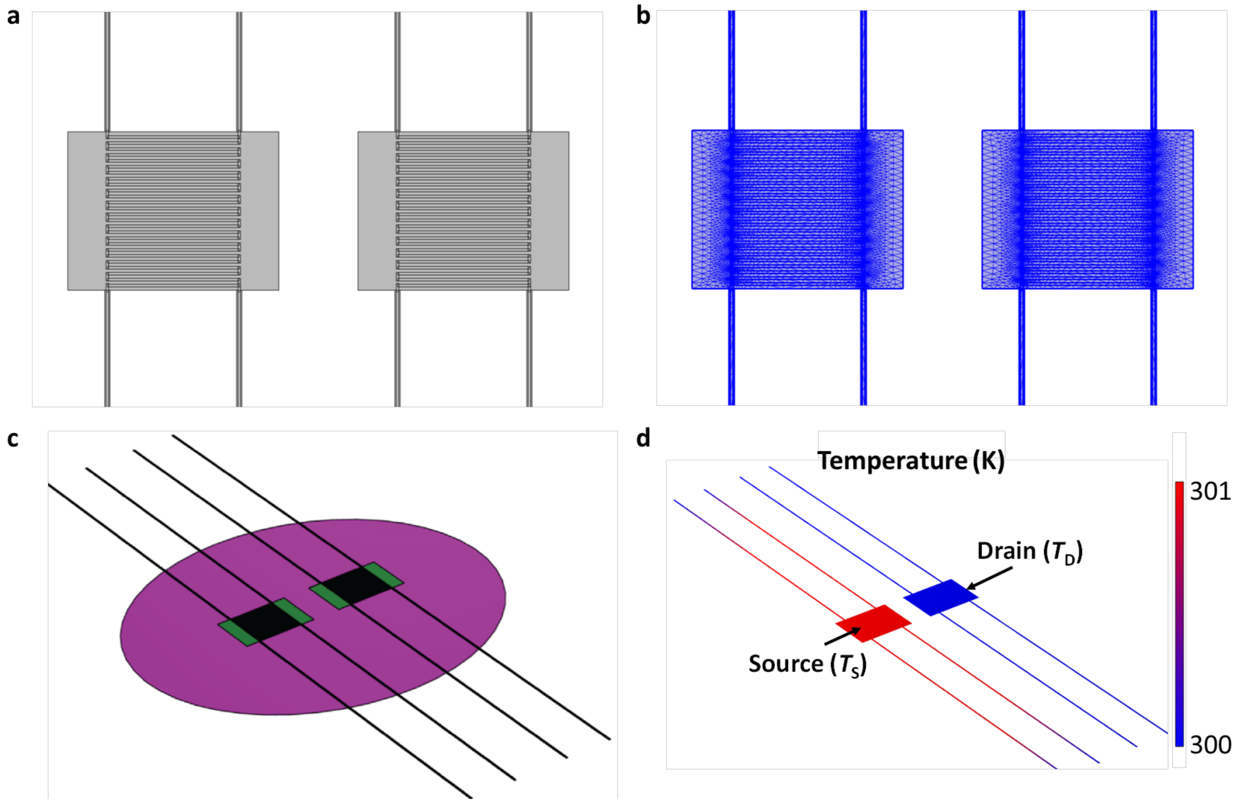

Supplementary Figure 7. Illustrations from FEM simulations were performed to estimate $G_{blackbody}$. **a,** Top view of the suspended device geometry as modeled in COMSOL. SiN membrane is dark green, Pt is light gray. **b,** Discretization mesh on the suspended membrane structures. **c,** The complete geometry used in the simulation, including the VOx-covered substrate of the gate, is shown in purple. **d,** Computed surface temperatures of the source membrane and drain membrane.

Supplementary Note 8. Displacement of the gate device due to temperature change.

To determine the physical displacement of the gate when the gate device transitions into a metallic phase, FEM analysis (COMSOL Multiphysics) was performed. To calculate the maximum displacement under the condition of the highest temperature reached during the measurement, a constant temperature of 380 K was applied to the mesa surface, while one end of the two beams was maintained at room temperature. Supplementary Figure 8 shows the displacement of the gate along the *z*-axis, indicating that the largest displacement is less than 10 nm. This indicates that the displacement of the gate device is negligible compared to the gap distance (*d*), even when the gate temperature reaches up to 380 K.

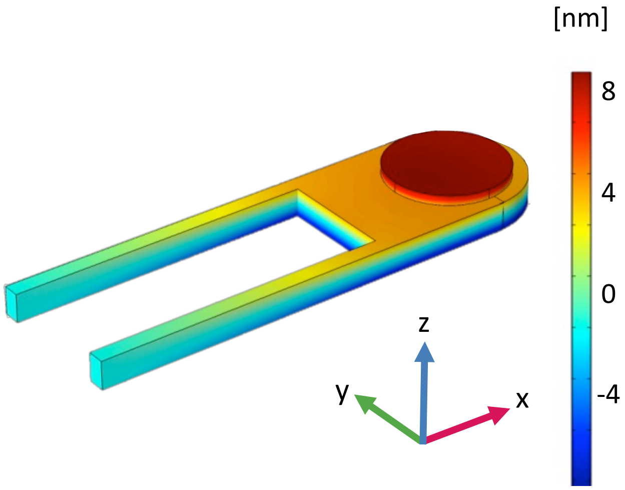

Supplementary Figure 8. Displacement of the gate device along the *z*-axis when the gate device is heated up to 380 K as modelled with COMSOL.

Supplementary Note 9. Comparison of total power flowing from source to drain between experiments and calculations.

Here we compare the total power that is transferred from the source to the drain in our thermal transistor between the experiments and the calculations. In our SCUFF-EM model, we compute the power by providing a temperature differential of ~4.9 K, which corresponds to the applied temperature differential in the experiments. As can be seen, the experiments and the simulation agree qualitatively well with each other, but they quantitatively differ by about 30–40%. The differences are likely due to ignoring the contributions of supporting beams and uncertainty in the dielectric functions, as stated in the manuscript. The results for when the gate is in its 'metallic' state and when it is in its 'insulating phase' are shown below in Supplementary Figures 9a, b respectively.

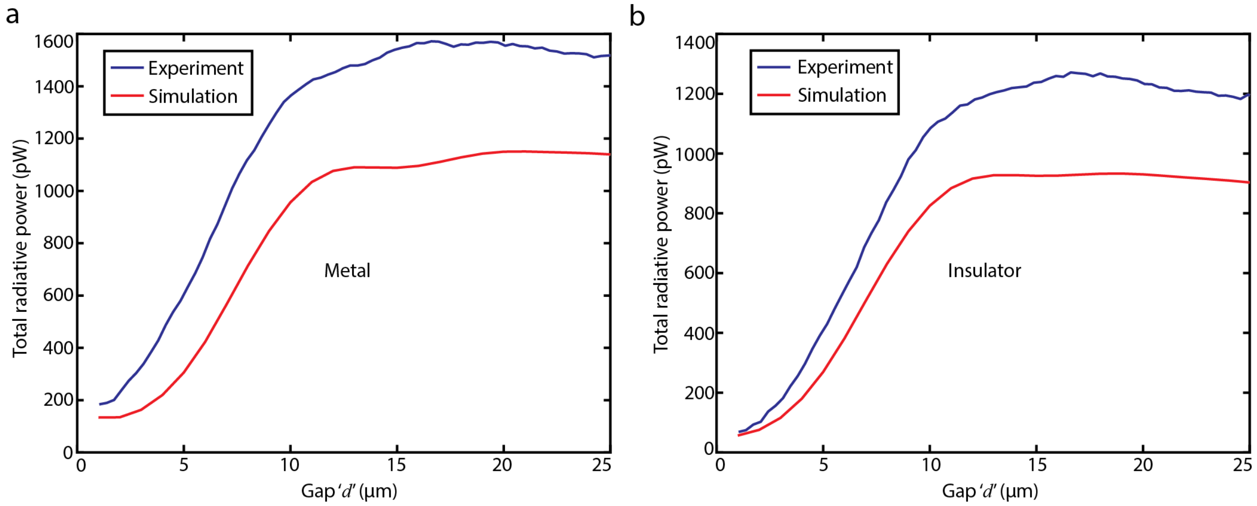

Supplementary Figure 9. Comparison of total power radiated from the source to the drain between the experiments and simulations. **a**, Total radiative power *vs* gap '*d*' for the case when the gate is metallic, **b**, same as in 'a' but the gate is in its insulating phase.

References

1. Thompson, D., Zhu, L., Meyhofer, E. & Reddy, P. Nanoscale radiative thermal switching via multi-body effects. *Nat. Nanotechnol.* **15**, 99-104 (2020)
2. Zhang, Y., Xiong, W., Chen, W., Zheng, Y. Recent progress on vanadium dioxide nanostructures and devices: Fabrication, properties, applications and perspectives. *Nanomaterials* **338**, 11 (2021).
3. Thompson, D. *et al.* Hundred-fold enhancement in far-field radiative heat transfer over the blackbody limit. *Nature* **561**, 216-221 (2018)
